# Tylosin Dosage Adjustment Based on Allometric Scaling in Male Turkeys

**DOI:** 10.3390/antibiotics10091057

**Published:** 2021-08-31

**Authors:** Błażej Poźniak, Marta Tikhomirov, Kamila Bobrek, Paweł Jajor, Marcin Świtała

**Affiliations:** 1 Department of Pharmacology and Toxicology, Faculty of Veterinary Medicine, Wroclaw University of Environmental and Life Sciences, ul. Norwida 31, 50-375 Wrocław, Poland; marta.tikhomirov@upwr.edu.pl (M.T.); pawel.jajor@upwr.edu.pl (P.J.); marcin.switala@upwr.edu.pl (M.Ś.); 2 Department of Epizootiology and Clinic of Birds and Exotic Animals, Faculty of Veterinary Medicine, Wroclaw University of Environmental and Life Sciences, pl. Grunwaldzki 45, 50-366 Wrocław, Poland; kamila.bobrek@upwr.edu.pl

**Keywords:** turkeys, allometric scaling, pharmacokinetics, tylosin, dose optimization, poultry, internal exposure

## Abstract

Turkeys’ body weight (BW) increases 10-fold within only 2.5 months, leading to a change in the pharmacokinetics (PK) of drugs according to allometric principles. Thus, the same dosage may lead to age-dependent variability in efficacy, in particular, to treatment failure and/or selection for resistance. The study aimed to investigate whether a non-linear dosage based on a published allometric model for tylosin clearance, may optimize the internal exposure in growing turkeys. The single dose PK study was performed on turkeys aged 6, 9.5, 13 and 17 weeks (BW from 1.75 kg to 15.75 kg). Tylosin was administered intravenously (i.v.) or orally (p.o.) according to following protocols: Dose = 31.6 × BW^0.58^ or Dose = 158 × BW^0.58^, respectively. Plasma tylosin was measured using high-performance liquid chromatography and non-compartmental PK analysis was performed. The area under the curve (AUC_last_) after i.v. administration was 8.90 ± 1.01; 7.51 ± 1.11; 6.54 ± 1.20 and 8.01 ± 1.75 mg × h/L in 6-; 9.5-; 13- and 17-week-old turkeys, respectively. After p.o. administration AUC_last_ was 4.80 ± 2.92; 4.60 ± 2.45; 3.00 ± 1.49 and 3.24 ± 2.00 mg × h/L in respective age groups indicating high variability. For i.v. administration, the non-linear dosage allowed to minimize the age-dependent variability in AUC. However, due to low oral bioavailability (8–12%) and resulting interindividual variability, the proposed approach may not improve tylosin efficacy in turkeys under farm conditions.

## 1. Introduction

The common assumption for veterinary antimicrobial use is the fixed dosage regimen for a given species. This simplification ignores the tremendous effects of certain physiological changes that develop over the lifespan of an individual (e.g., growth) on the pharmacokinetics (PK) of drugs [1]. Our team has systematically studied how the disposition of antimicrobials changes as broiler chickens or turkeys rapidly gain weight. For instance, in the heavy breeds of turkeys a 10-fold increase in the body weight (BW) is seen within just 2.5 months [2]. This outstanding pace of weight gain, much desired by the animal production industry, is not occurring without a certain physiological cost, particularly to the circulatory system and hemodynamics. It was shown in both turkeys and broiler chickens that the relative cardiac output drops gradually during the short period of intensive growth [2,3]. The sudden increase in the total mass of muscles and their growing metabolic demands translate directly to the lower fraction of blood perfusing other organs, e.g., liver and kidney. As a result, a significant age-dependent decrease in the capability of the clearing organs to eliminate drugs is seen. Indeed, the difference between the total body clearance (CL) in different age groups may be even three-fold as shown for metronidazole [2] or enrofloxacin [4] in turkeys. Significant age-dependent drop in CL has also been described for florfenicol in broilers [3] as well as for amoxicillin [5], doxycycline [6] and sodium salicylate [7] in turkeys. Recently, our team has reported that this effect is also seen for tylosin [8]. Between the 5th and the 16th week of age, the mean value of CL for tylosin dropped from 3.81 L/h/kg to only 1.42 L/h/kg leading to a vast difference in the internal exposure to the drug in young turkeys as compared to the mature ones [8].

For antimicrobials, the consequences of this change may be profound as the same drug dose (relative to BW) will result in different effects in birds of different age. In young birds which clear the drug faster, there is a high risk of subtherapeutic concentrations, treatment failure and selection of resistant strains of bacteria [4]. This may contribute to the higher incidence of antimicrobial resistance reported in young birds [9]. It is known that antimicrobial efficacy depends on reaching sufficient values of the integrated pharmacokinetic-pharmacodynamic (PK/PD) parameters [10,11]. For many antimicrobials, the effect correlates with the area under the (free fraction) concentration-time curve (AUC, the measure of the internal exposure to the drug) divided by the minimum inhibitory concentration (MIC) for a pathogen of interest [12]. Since AUC is directly dependent on CL and dose, based on the equation:

CL = Dose/AUC,(1)

it seems logical that a three-fold difference in CL should be compensated by a three-fold change in the dose in order to sustain the same antimicrobial efficacy (considering the constant MIC). It is widely acknowledged that the emergence of antimicrobial resistance obliges the veterinary community to develop and optimize tools that will allow for more prudent use of antimicrobials, limited to precise strikes only when needed and only at the right dose [13]. We believe that evidence-based flexible dosage protocols should supersede the fixed dose paradigm as the veterinary response to precision medicine—such as precise drug dosage at the farm scale.

Since the age-dependent change in CL follows the power-law relationship with BW and is quite predictable in broilers and turkeys, the mathematical function describing this relation may be easily incorporated into the non-linear dosage protocol following the concept of intra-species scaling of the dose [3]. In a series of recent papers we developed a non-linear dosage protocol for enrofloxacin [4] and later tested it in turkeys [14] to assess if the age-related variability in the internal exposure to the drug may be eliminated by this approach. It was found that the non-linear dosage allowed to decrease this variability by more than half for the intravenous (i.v.) administration and by almost 40% for the oral route. It is not known, however, whether these excellent results may be reproduced for other drugs as well.

Therefore, the aim of this study was to assess the applicability of the intra-species scaling of the dose using a non-linear dosage protocol for tylosin in turkeys. Since enrofloxacin is characterized by efficient oral absorption and renal elimination, in the current study we decided to test this approach with tylosin—a very different drug with relatively low bioavailability and predominately eliminated with the bile. It is a broad-spectrum time-dependent macrolide antibiotic frequently used in companion and farm animals. It inhibits bacterial protein synthesis that leads to bacteriostatic effect [15]. Typical indications include infections caused by *Lawsonia intracellularis* in pigs as well as *Bordetella* sp., *Pasteurella multocida* and *Mycoplasma* spp. in a wide range of animal species [16,17,18]. In poultry, primary indications include chronic respiratory disease (CRD) in chickens and infectious sinusitis in turkeys caused by *Mycoplasma gallisepticum* as well as joint and respiratory disease caused by *Mycoplasma synoviae* [19]. The non-linear dosage protocol used in this study was theoretically developed in our recently published paper [8]. In this previous study, tylosin was administered according to the standard linear dosage of 10 and 50 mg/kg for i.v. and oral administration, respectively. This allowed to quantitate the age-dependent change in tylosin CL in turkeys and to develop an allometric model predicting this change. The allometric exponent describing the relationship between tylosin CL and BW as well as the normalizing factor for the expected AUC (allometric coefficient) were used to develop the following non-linear dosage protocols that were utilized in this current study: for i.v. administration Dose = 31.6 × BW^0.58^ and for the oral administration Dose = 158 × BW^0.58^ [8].

## 2. Results

**Figure 1:**
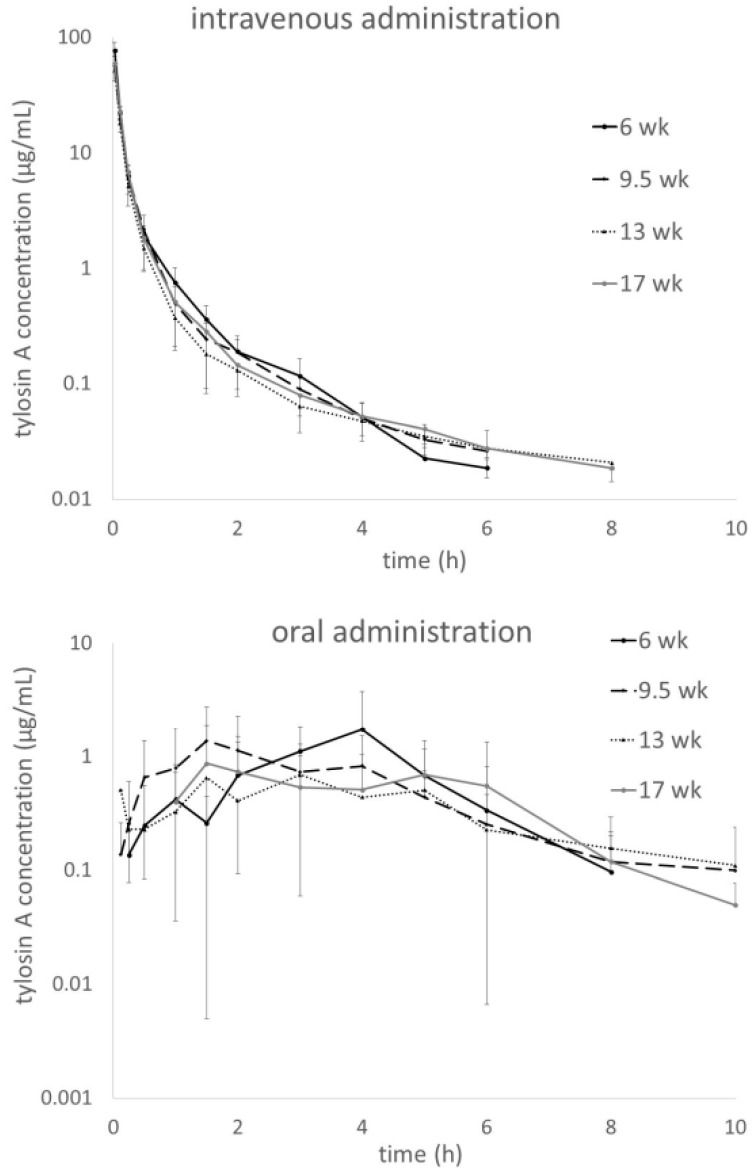
Plasma tylosin A concentrations after single intravenous (upper panel) and oral dose (lower panel) of tylosin tartrate calculated according to the protocols: Dose = 31.6 × BW^0.58^ or Dose = 158 × BW^0.58^ for the intravenous and oral administration, respectively.

Plasma tylosin concentration-time plots obtained in turkeys at four different ages are shown in Figure 1. The upper panel shows the i.v. and the lower one the oral (p.o.) administration. The mean curves for the age groups were overlaid for better visualization of age- and dose-dependent changes in profiles. For i.v. administration, the distribution phase turns into the elimination phase without a clearly identifiable border and all four curves look similar. For the oral administration there is no distinct peak and high inter-individual variability precludes identification of clear trends.

**Figure 2:**
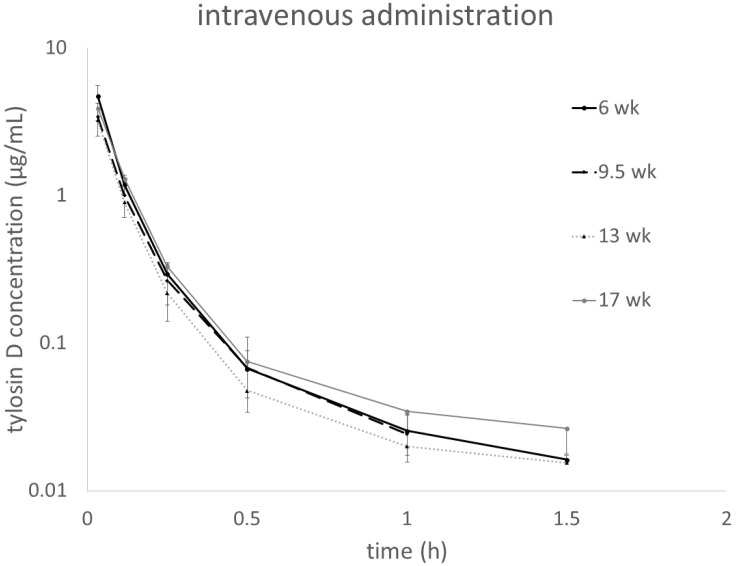
Plasma tylosin D concentrations after single intravenous dose of tylosin tartrate calculated according to the protocol: Dose = 31.6 × BW^0.58^.

Figure 2 shows plasma tylosin D concentrations assessed in parallel to the tylosin A measurements in turkeys administered i.v. with tylosin tartrate. For oral administration, the concentrations of the metabolite were very low and quantifiable only at random time points in some individuals (data provided in the Supplementary File S1).

Pharmacokinetic parameters obtained after i.v. tylosin tartrate administration are presented in Table 1.

There is a statistically significant difference in the AUC values between the 6-week-old and 13-week-old turkeys but the higher value for the 17-week-old individuals does not support it as a trend. Variability within age groups (as represented by CV_group_) increases with age, however, the measure of variability for the pooled AUC values (CV_pooled_) is lower than the group value for the largest turkeys. The mean residence time (MRT) slowly increases to reach statistical significance when the youngest and the oldest birds are compared. This is paralleled with a significant decrease in the volume of distribution (Vd_ss_) and CL, as well as the prolongation of the elimination half-life (T_1/2el_). The initial concentration (C_init_) was the highest in the youngest individuals but no clear age-dependent trend was observed. Tylosin D paralleled the changes in the PK of tylosin A and no clear age-related trends were seen. The relative exposure to tylosin D expressed in the AUC ratio was approx. 5% in all turkeys. For the oral administration, the PK parameters are presented in Table 2.

The AUC values show high variability in all age groups with CV_group_ values ranging from 49.7 to 61.7%. The CV_pooled_ value of 59.7% is similar to the within-group measures of variability. Most of the other parameters show similarly high degree of variability and no statistical significance of differences was seen in the four age groups. Bioavailability was found to be around 10% and, similarly to the parameters of absorption, seems unaffected by the age. In a number of individuals, it was not possible to identify a clear slope of the elimination phase so the parameters extrapolated to infinity as well as T_1/2el_ were not calculated for these turkeys. This effect was occasional in younger animals (*n* = 2 in 6 week and 9.5 week old turkeys) and more common in the older ones (*n* = 3 and *n* = 6 in 13 week and 17 week-old turkeys, respectively). Raw data for individual plasma drug concentrations and PK parameters are provided in the supplementary files S1 and S2, respectively.

## 3. Discussion

The amount of scientific evidence supporting the role of growth in the change of antimicrobial PK in broiler chickens and turkeys is extensive [2,3,4,5,6,7]. It is clear that the internal exposure to antimicrobials in these birds changes according to the power law relation to BW [20,21,22] and may be responsible for the suboptimal response to treatment in young individuals [4]. Studies on non-linear dosage of enrofloxacin in turkeys showed that taking into account the age-dependent change in CL caused a more than two-fold decrease in interindividual variability in AUC [14] as compared to traditional dose calculation based on linear relation to BW (10 mg/kg) [4]. These studies suggest that age-dependent change in PK is the most important source of interindividual variability in the internal exposure to enrofloxacin in turkeys and, as a result, that the young individuals may be systematically underdosed. This is a classical condition that facilitates the selection of resistant bacterial strains [23]. There is, indeed, evidence that antimicrobial-resistant bacteria are more frequently isolated from younger birds [9]. It should be stressed that the primary aim of the therapeutic intervention with antimicrobials should be pathogen eradication and not only the control of clinical disease [10]. The presence of a subpopulation of animals as pathogen reservoir in the flock may easily lead to pathogen persistence as well as to the selection of resistant mutants and reappearance of the mass disease. As a result, drug administration may have to be repeated and the overall antimicrobial consumption will be high. This could be avoided by the precise use of adequate doses at the beginning of the disease to eradicate the pathogens from the whole flock [13]. Such successful intervention may be possible only if the most relevant sources of variability in the animals’ internal exposure to drugs are identified and accounted for. For instance, one study argued that circadian variation in the uptake of medicated feed or water in chickens may be a cause of suboptimal internal exposure to tylosin at night [24]. Although the interpretation of data and recommendations presented by these authors have been criticized [25], this indicates that precise tylosin administration in poultry draws scientific attention. The current cutting edge approach based on population pharmacokinetic modelling allows to assess and quantify the sources of variability [12]. Coupled with the Probability of Target Attainment analysis it allows one to assess the dose that has to be administered to ascertain that, e.g., 90% of the treated population will have a satisfactory internal exposure to the drug [12]. However, if treatment is still based on the linear dosage (i.e., expressed in mg/kg), it may mean that a large proportion of the flock will be treated with an unnecessary high dose. Therefore, we hypothesize that in case of rapidly growing poultry lines where the variability in CL is well defined and predictable, a simple approach based on intra-species allometric scaling of the dose may be very efficient in improving the prudent use of antimicrobials and also easy to apply on the farm level.

For enrofloxacin, the inclusion of allometric change in CL into the dosing protocol allowed to minimize variability in AUC, however, it is not certain if this would be the case in other drugs. It is often assumed that drugs eliminated by the kidneys should perform best in allometric scaling [26]. However, the results of the current study suggest that the route of elimination may be of lesser importance than expected. Tylosin is primarily eliminated with bile in many mammalian species like dogs and camels [27,28]. A recent study in sheep suggests that in some species urinary excretion may also play a role [29]. It is assumed that biliary elimination is also important in birds [30], but the dominating route of elimination in birds is not well evidenced. In the current study we have shown that the application of a non-linear allometric tylosin dosage for i.v. administration allowed to keep the variability in AUC (CV_pooled_) at a very low level of 19.6% which is similar to the interindividual variability seen in the specific age groups (CV_group_, Table 1). This value is much lower compared to the CV_pooled_ of 45.3% found in our recent study when tylosin was administered according to standard linear dose of 10 mg/kg [8]. This astonishing improvement suggests that allometric change in CL is indeed the most important source of variability in drug exposure when elimination is the only determinant of the PK, as it is in the i.v. administration. The residual variability of 19.6% is very low thanks to very high genetic homogeneity seen in BUT-9 turkeys and other meat poultry lines and results from a sum of minor physiological differences (e.g., hydration status, liver and kidney function) and unavoidable experimental or analytical error.

For clinical application, however, this reduction in interindividual variability should also hold for the oral treatment which is the primary route of administration under farm conditions. Unfortunately, for the oral route the improvement in CV_pooled_ for AUC was only minor: 59.7% with the non-linear dosage vs. 68.5% for the linear dosage of 50 mg/kg obtained in the earlier study [8]. This difference between CV values for the two routes of administration can be explained as the introduction of an additional layer of variability in the oral administration due to the process of absorption. Apparently, this source of variability is of major importance as indicated by the CV_group_ values for turkeys of the same age. They are very high, and similar to the pooled value, even despite the fact that the age-effect does not contribute to CV_group_. Therefore, the interindividual variability seems to obscure the allometric relationship seen in the i.v. administration. Why is it so? In contrast to enrofloxacin, which has very high bioavailability [4,31,32], tylosin is only poorly absorbed from the gastrointestinal system [8]. As can be seen in Figure 1, there is no distinct peak indicating a clear separation of the absorption and elimination phases. Additionally, it can be appreciated from Table 1, that the T_max_ is highly variable and the F value is only 8–12%. As it has been elegantly explained by Hellriegel et al. [33], the lower the bioavailability, the higher the inter-subject variability in this parameter and, as a consequence, in the internal exposure. Therefore, intra-species allometric scaling of a dose is a method that may be considered only for drugs with high bioavailability (as shown for enrofloxacin), whereas the route of elimination seems to be of lesser importance. There is an additional reason to avoid the use of antimicrobials with low oral bioavailability for general infections. The dose required to reach significant internal exposure needs to be very high and the exposure of commensal microbiota of the gut and subsequent environmental contamination leads to a high risk of selection of resistant bacteria [34].

## 4. Materials and Methods

### 4.1. Animals

Turkeys (20 males, 3-week-old, line BUT-9) were provided by a commercial breeding facility. Only male turkeys were chosen to limit the sex-related differences in weight gain. The birds were housed in an animal house with ambient temperature of 20–23 °C, relative humidity of 50–60% and straw as the bedding material. Water and commercial feed (free of antimicrobial and antiprotozoal agents) were provided ad libitum. Two weeks were allowed for acclimatization before the start of the experiment. The birds were individually marked and randomly divided into two groups (*n* = 10 each): one group for an i.v. and the other group for the oral route of administration. The experiment was approved by the Local Animal Experimentation Committee in Wrocław, Poland (permit number 33/2016). All efforts were made to minimize animals’ suffering and to reduce the number of animals used. All procedures involving animals were performed in accordance with national and international laws and policies.

### 4.2. Pharmacokinetic Study

All individuals were used in a single-dose PK studies four times that is when the birds reached the age of 6; 9.5; 13 and 17 weeks. At this age, the turkeys had 1.75 ± 0.13; 4.93 ± 0.46; 9.31 ± 0.54 and 15.75 ± 1.04 kg BW, respectively. The studies were carried out according to a parallel design with four phases and two groups differing by the route of administration, without blinding. Before each experiment, the birds’ health was checked by physical examination. All animals remained healthy throughout the study. Tylosin tartrate (pharmaceutical grade, VETOS-FARMA, Poland) was administered i.v. into vena brachialis or orally as a gavage into the crop at a single dose calculated based on the previously described dosing protocols: Dose = 31.6 × BW^0.58^ or Dose = 158 × BW^0.58^, respectively. This protocol has been developed based on allometric scaling. The CL may be interpreted as the proportionality factor between the dose (D) and the AUC (assuming first-order kinetics):

D = CL × AUC(2)

The AUC is expected to be a constant value for the constant AUC/MIC related to a given pathogen so D becomes directly proportional to CL. In the recent work we have shown that the relation between CL and BW is not linear but follows the power law relation with the exponent of 0.58. Therefore, the power law structure and the exponent for CL also applies to the dose:

D = cBW^0.58^ (3)

If the AUC value obtained for the largest turkeys in the previous study is considered satisfactory (to provide the optimal AUC/MIC), then the coefficient c is calculated as follows:

c = D/(BW^0.58^) (4)

by putting in the mean dose of tylosin for the heaviest individuals (150 mg) and their mean body weight (15 kg). This provides with the following tylosin dosage protocol in turkeys:

D = 31.6 BW^0.58^ (5)

This dosage was expected to ensure a constant AUC of around 7 mg × h/L (mean for the heaviest individuals in the recent study) in turkeys irrespective of age and BW. For the oral administration, the initial dose was 5 times higher than for the i.v. administration, therefore the coefficient c is 5 times higher (158). In the i.v. part of the current study, the dosage administered according to the equation above was 24.9; 16.2; 12.4 and 9.9 mg/kg in turkeys weighing 1.76; 4.92; 9.22 and 15.76 kg, respectively. The oral dose was 125.2; 80.9; 61.7 and 49.7 mg/kg in turkeys weighing 1.74; 4.93; 9.40 and 15.73 kg, respectively. All animals had been fasted for 10 h before drug administration and the experiments commenced at 7:00 A.M. The i.v. injection lasted 1 min. After that, blood samples (1 mL) were collected from jugular vein into heparinized syringes before the experiment as well as at 2; 7; 15; 30 min and 1; 1.5; 2; 3; 4; 5; 6; 8; and 10 h after drug administration (to minimize the unnecessary blood loss in the youngest individuals, samplings at 8 and 10 h were skipped in this group). For the p.o. studies, the first blood sample was taken at 7 min and since 15 min all sampling times were identical as in the i.v. study (sampling at 10 h was skipped in the youngest turkeys). After centrifugation (10 min, 3000× *g*) plasma samples were stored at −70 °C until assayed.

### 4.3. Determination of Tylosin A and D in Plasma

Plasma concentrations of tylosin A and D were measured by an in-house developed high-performance liquid chromatography (HPLC) method as described in our recent paper [8]. Briefly, a Waters Alliance HPLC system (Waters, Milford, MA, USA) equipped with a 2996 PDA detector and a Hypersil GOLD aQ (5 µm) 150 mm × 4.6 mm column (Thermo Fisher Scientific, Waltham, MA, USA) was used to separate and quantify both compounds. The mobile phase comprised 70% 0.02 M KH_2_PO_4_ (Sigma-Aldrich, Tokyo, Japan) at pH 2.4 and 30% acetonitrile (J.T. Baker, USA) and was set at a flow rate of 1 mL/min. Tylosin A and D were detected by UV absorption at 286 nm. Retention time for tylosin A was 10.5 min and for tylosin D it was 8.2 min. Plasma samples (0.5 mL) were extracted with 1.5 mL ethyl acetate for 15 min. For better separation of phases, samples were centrifuged (37,000× *g*, 4 °C, 15 min) and frozen in −70 °C. After that, supernatant was collected and dried in vacuum at 45 °C. Dry residues were reconstituted in 250 µL of water and transferred to autosampler vials. Plasma concentrations of tylosin A and D were calculated based on calibration curves prepared in blank plasma spiked with analytical standards (Sigma-Aldrich, Taufkirchen, Germany). Linearity, specificity, recovery, inter-assay and intra-assay coefficient of variation (CV) were assessed. For tylosin A, the limit of detection (LOD) was 0.003 µg/mL and the limit of quantification (LOQ) was 0.011 µg/mL. For tylosin D, LOD was 0.034 µg/mL and LOQ was 0.104 µg/mL. Assay validation for tylosin A and tylosin D (at a concentration of 3.12 µg/mL for tylosin A and 0.625 µg/mL for tylosin D) indicated an intra-assay coefficient of variation (CV) of 3.4% and 3.0%, respectively. An inter-assay CV for tylosin A was 6.4% and for tylosin D was 9.4%. The recovery rate for tylosin A and D was 102% and 158%, respectively (at 6.25 µg/mL).

### 4.4. Pharmacokinetic Analysis

The PK parameters for tylosin A and tylosin D were calculated based on non-compartmental approach (TP4.1 software, ThothPro, Gdańsk, Poland). For the i.v. administration, the area under the concentration-time curve from time 0 to the last sampling (AUC_last_) and to infinity (AUC_inf_), the area under first moment curve from time 0 to infinity (AUMC_inf_), mean residence time (MRT_inf_), body clearance (CL), apparent volume of distribution at steady state (Vd_ss_), elimination half-life (T_1/2el_) and the initial concentration (C_init_) were determined. For the calculation of T_1/2el_, at least three last datapoints from the linear portion of the terminal slope have been used. For the p.o. study, AUC_last_, AUC_inf_, AUMC_last_, AUMC_inf_, MRT_last_, MRT_inf_, T_1/2el_ as well as peak plasma concentration (C_max_) and the time when it was observed (T_max_) were assessed. Mean absorption time (MAT) after oral administration was calculated as follows: MAT = mean MRTp.o.—mean MRTi.v. The bioavailability (F) of orally administered drug was calculated as follows: F (%) = 100 × [(mean AUC_inf_p.o. × Dose i.v.)/(mean AUC_inf_i.v. × Dose p.o.)]. For tylosin D, basic PK parameters (AUC_last_, C_max_) are provided only for the i.v. administration of tylosin tartrate. After oral administration, only a few individuals were showing quantifiable levels of tylosin D and these were seen at rather random times precluding any credible calculation of PK parameters. To explore the potential age-dependent differences in tylosin A metabolism, the ratio of AUC_last_ for tylosin A and D has been determined.

### 4.5. Statistical Analysis of Pharmacokinetic Parameters

The normality of distribution of the PK parameters was assessed by the Shapiro-Wilk test (Statistica 13.3, Tibco, Palo Alto, CA, USA). The parameters were found to be normally distributed and they are presented as mean and standard deviation (±SD). Statistical significance of the differences was assessed by one-way ANOVA with post-hoc Tukey test. Differences with *p* < 0.05 were considered significant. To assess the sources of variability in the internal exposure, the CV was calculated as standard deviation/mean for the AUC_inf_ within the age group (inter-individual variability, age-independent) as well as for the pooled values for all the age groups (age-dependent variability).

## 5. Conclusions

It is concluded that intra-species allometric scaling of the dose does not decrease high variability in internal exposure to tylosin in growing turkeys. The reason for that is poor oral absorption of this drug, therefore, the allometric approach to dosage should be limited to drugs with excellent oral bioavailability. As shown for tylosin, the route of elimination may be of lesser importance as a predictor for the outcome of intra-species scaling of the dose.

## Figures and Tables

**Table 1:** Pharmacokinetic parameters after single intravenous tylosin tartrate administration to turkeys dosed according to the protocol: Dose = 31.6 × BW^0.58^.

Parameter		Unit	Age Groups (Body Weight ± SD), *n* = 10 Each			
			6 Weeks (1.76 ± 0.11 kg)	9.5 Weeks (4.92 ± 0.40 kg)	13 Weeks (9.22 ± 0.56 kg)	17 Weeks (15.76 ± 1.10 kg)
TYL A	AUC_last_	mg × h/L	8.90 ± 1.01 ^a^	7.51 ± 1.11 ^ab^	6.54 ± 1.20 ^b^	8.01±1.75 ^a^
	CV_group_	%	11.4	14.8	18.3	21.9
	CV_pooled_	%	19.6			
	AUC_inf_	mg × h/L	8.93 ± 1.01 ^a^	7.58 ± 1.12 ^ab^	6.62 ± 1.22 ^b^	8.10 ± 1.75 ^a^
	AUMC_inf_	mg × h^2^/L	3.02 ± 0.59	2.99 ± 0.70	2.86 ± 1.23	3.72 ± 1.29
	MRT_inf_	h	0.34 ± 0.05 ^a^	0.39 ± 0.07 ^ab^	0.42 ± 0.13 ^ab^	0.46 ± 0.12 ^b^
	CL_(rel)_	L/h/kg	2.83 ± 0.37 ^a^	2.18 ± 0.30 ^b^	1.94 ± 0.33 ^b^	1.29 ± 0.32 ^c^
	Vd_ss(rel)_	L/kg	0.87 ± 0.12 ^a^	0.71 ± 0.16 ^ab^	0.59 ± 0.17 ^b^	0.42 ± 0.10 ^c^
	T_1/2el_	h	1.25 ± 0.53 ^a^	1.64 ± 0.58 ^ab^	2.27 ± 1.06 ^ab^	2.68 ± 1.20 ^b^
	C_init_	µg/mL	77.6 ± 12.8 ^a^	56.3 ± 12.4 ^b^	52.9 ± 11.3 ^b^	60.0 ± 10.8 ^b^
TYL D	AUC_last_	mg × h/L	0.44 ± 0.06 ^a^	0.34 ± 0.07 ^ab^	0.31 ± 0.07 ^b^	0.42 ± 0.10 ^a^
	C_max_	µg/mL	4.73 ± 0.84 ^a^	3.42 ± 0.81 ^b^	3.24 ± 0.73 ^b^	3.89 ± 0.71 ^ab^
AUC_D_/AUC_A_		%	4.91 ± 0.39 ^ab^	4.55 ± 0.38 ^a^	4.67 ± 0.48 ^a^	5.29 ± 0.50 ^b^

Values expressed as mean ± SD. Abbreviations: BW—body weight, TYL A—tylosin A, TYL D—tylosin D, AUC_last_—area under the curve from time 0 to the last quantifiable value, CV_group_—coefficient of variation of AUC_last_ within the age group, CV_pooled_—coefficient of variation of AUC_last_ for all age groups, AUC_inf_—area under the curve from time 0 to infinity, AUMC_inf_—area under the first moment curve, MRT—mean residence time, CL_(rel)_—relative body clearance, Vd_ss(rel)_—relative volume of distribution at steady state, T_1/2el_—elimination half-life, C_init_—initial concentration, AUC_D_/AUC_A_—the ratio of AUC_last_ for the metabolite and the parent compound. Values in a row not sharing a common superscript letter are statistically different, *p* < 0.05. Lack of superscript indicates lack of statistical difference (CV values were not statistically comparable).

**Table 2:** Pharmacokinetic parameters after single oral tylosin tartrate administration to turkeys dosed according to the protocol: Dose = 158 × BW^0.58^.

Parameter		Unit	Age Groups (Body Weight ± SD), *n* = 10 Each			
			6 Weeks (1.74 ± 0.13 kg)	9.5 Weeks (4.93 ± 0.49 kg)	13 Weeks (9.40 ± 0.48 kg)	17 Weeks (15.73 ± 0.91 kg)
TYL A	AUC_last_	mg × h/L	4.80 ± 2.92	4.60 ± 2.45	3.00 ± 1.49	3.24 ± 2.00
	CV_group_	%	60.9	53.4	49.7	61.7
	CV_pooled_	%	59.7			
	AUC_inf_ *	mg × h/L	5.25 ± 3.13	5.28 ± 2.25	3.53 ± 1.42	4.92 ± 1.72
	AUMC_last_	mg × h^2^/L	18.07 ± 12.10	15.16 ± 6.85	11.64 ± 7.41	12.36 ± 8.71
	AUMC_inf_ *	mg × h^2^/L	19.42 ± 13.28	16.71 ± 5.24	13.48 ± 8.11	19.75 ± 6.17
	MRT_last_	h	3.67 ± 0.74	3.64 ± 1.39	3.83 ± 1.32	3.65 ± 1.40
	MRT_inf_ *	h	3.58 ± 0.55	3.50 ± 1.33	3.67 ± 1.06	4.10 ± 0.91
	MAT **	h	3.36	3.32	3.52	3.31
	T_1/2el_ *	h	1.31 ± 1.18	1.37 ± 0.82	1.13 ± 0.35	1.04 ± 0.25
	C_max_	µg/mL	2.45 ± 1.63	1.83 ± 1.16	1.34 ± 0.79	1.35 ± 0.91
	T_max_	h	3.50 ± 1.27	3.00 ± 1.68	2.41 ± 1.45	3.15 ± 1.72
	F **	%	10.9	12.2	9.1	8.1

Values expressed as mean ± SD. Abbreviations: BW—body weight, TYL A—tylosin A, AUC_last_—area under the curve from time 0 to the last quantifiable value, CV_group_—coefficient of variation of AUC_last_ within the age group, CV_pooled_—coefficient of variation of AUC_last_ for all age groups, AUC_inf_—AUC from time 0 to infinity, AUMC_inf_—area under the first moment curve, AUMC_last_—AUMC from time 0 to the last quantifiable value, MRT_inf_—mean residence time from time 0 to infinity, MRT_last_—MRT from time 0 to the last quantifiable value, MAT—mean absorption time, T_1/2el—_elimination half-life, C_max_—maximal concentration, T_max_—time when the C_max_ is reached, F—bioavailability. * Due to inability to estimate the terminal slope in some individuals, parameters extrapolated to infinity and T_1/2el_ were obtained for a limited number of individuals: 6 week, *n* = 8; 9.5 week, *n* = 8; 13 week, *n* = 7; 17 week, *n* = 4. ** Calculated for the mean value from the i.v. administration and the mean value from oral administration. None of the differences were found to be statistically significant (*p* < 0.05). Values of CV, F and MAT are statistically incomparable.

## Data Availability

All the raw data are available in the Supplementary Material published with this paper.

## Supplementary Materials

The following are available online at https://www.mdpi.com/article/10.3390/antibiotics10091057/s1, File S1: Individual pharmacokinetic parameters for tylosin oral and intravenous administration in turkeys; File S2: Individual concentrations of tylosin A and tylosin D in turkeys.
